# Experiencing visual impairment in a lifetime home: an interpretative phenomenological inquiry

**DOI:** 10.1007/s10901-017-9553-6

**Published:** 2017-05-19

**Authors:** Clíona Rooney, Karim Hadjri, Keith Mcallister, Máirín Rooney, Verity Faith, Cathy Craig

**Affiliations:** 1 School of Planning, Architecture and Civil Engineering, Queen’s University Belfast, David Keir Building, Stranmillis Road, Belfast, UKBT9 5AG UK; 2 Present Address: MUSSI, Iontas Building, Maynooth University, Co. Kildare, Ireland; 3 Sheffield School of Architecture, The University of Sheffield, Arts Tower, Western Bank, Sheffield, S10 2TN UK; 4 School of Natural and Built Environment, Queen’s University Belfast, David Keir Building, Stranmillis Road, Belfast, UKBT9 5AG UK; 5 St. Angela’s College (National University of Ireland Galway), Lough Gill, Sligo, Ireland; 6 School of Psychology, Queen’s University Belfast, David Keir Building, Stranmillis Road, Belfast, BT7 1NN UK

**Keywords:** Ageing, Housing, Interpretative phenomenological analysis, Lifetime homes, Visual impairment

## Abstract

Lifetime home standards (LTHS) are a set of standards aimed at making homes more accessible. Previous research, however, indicates that LTHS do not adequately meet the needs of those with sensory impairments. Now, with visual impairment set to increase globally and acknowledging the recognised link between quality of dwelling and wellbeing, this article aims to examine the experiences of visually impaired people living in lifetime homes. The objectives are to investigate existing lifetime homes and to identify whether LTHS meet occupants’ needs. Qualitative semi-structured interviews were carried out with six visually impaired people living in homes designed to LTHS in Northern Ireland. Collected data was analysed using interpretative phenomenological analysis identifying three super-ordinate themes: (1) living with visual impairment; (2) design considerations and (3) coping strategies. A core theme of balance between psychological and physical needs emerged through interconnection of super-ordinate themes. Although there are benefits to living in lifetime homes, negative aspects are also apparent with occupants employing several coping strategies to overcome difficulties. Whilst residents experience negative emotions following visual impairment diagnoses, results suggest that occupants still regard their homes as key places of security and comfort in addition to then highlighting the need for greater consideration of specific individual needs within general guidelines.

## Introduction

World populations are becoming increasingly older with average life expectancy increasing by twenty years since 1950. This is now predicted to extend by another 10 years by 2050 (United Nations 2002). Walford and Kurek (2008) assert that population ageing is now the main demographic process in the European Union (EU) where numbers of people aged over 60 are rising by more than 2 million every year (Eurostat 2010). Similarly, in Northern Ireland (NI), the number of adults aged over 65 is forecasted to increase by 63.5% between 2012 and 2032 (NISRA 2013). Furthermore in NI, the population of people aged over 85 is projected to increase by 19.6% between 2012 and 2017 (NISRA 2013).

With regard to visual impairment, older adults are most susceptible to eye disorders such as macular degeneration, cataract, glaucoma and diabetic retinopathy (Stuen and Faye 2003).

In the UK, the numbers of blind and partially sighted people are expected to rise from 1.79 million in 2010 to 4 million by 2050 with the population prevalence of visual impairment projected to rise from 3% in 2010 to 5.2% in 2050 (Access Economics 2009). NI Census, data show that 30,862 people declared themselves as blind or partially sighted (NIRSA 2013a, May). Proportionally, a greater percentage of older people are visually impaired than younger populations. For instance, 10% of people aged 25–44 are visually impaired compared to 59% of people aged 65 or older in NI (NIRSA 2013a, May). Additionally, Diabetes UK (2012) state that the percentage of people living with diabetes in NI has increased by 33% from 2007 to 2012. These statistics strongly suggest that in the future the numbers of visually impaired people will likely increase due to an ageing population coupled with conditions that contribute to sight loss.

When designing our built environment, it is important to consider the needs of a diverse population. However, twentieth century housing design mainly focused on able-bodied requirements (Imrie 1998). Architectural design standards met the needs of the “ideal” or average man and therefore atypical physique was not adequately represented (Gilson and DePoy 2011). As a response, LTHS were developed in the early 1990s by Joseph Rowntree Foundation (Lund 2011). They are based on the principle that homes should be accessible to everyone including older people, children and people with impairments (Irish Council for Social Housing 2006). Lifetime homes offer flexibility and can be easily adapted to meet occupants’ changing needs throughout life. LTHS are a set of 16 design criteria and focus on three main areas (Table 1): ease of approach to a home; circulation within a home and approach to key facilities (Goodman 2011). They consider features such as level access, width of doorways, circulation spaces, provision for future adaptations, access to floors above and height of controls in the home.

**Table 1:** Lifetime home standards (LTHS)

*Access*
1. Car Parking Widths
2. Approach to dwelling from parking
3. Approach to all entrances
4. Entrances
5. Communal stairs and lifts
*Inside the home*
6. Internal doorways and hallways
7. Circulation space
8. Entrance-level living space
9. Potential for entrance-level bed-space
10. Entrance-level WC and shower drainage
11. WC and bathroom walls
12. Stairs and potential for through-floor lift in dwellings
13. Potential for fitting of hoists and bedroom/bathroom relationship
14. Bathrooms
*Fixtures and fittings*
15. Glazing and window handle heights
16. Location of service controls

Although Census data show that 64% of visually impaired people in NI live in owner occupied housing, in comparison with the overall population, they are disproportionately represented in social housing^1^ (Russell 2013). Twenty-two per cent of blind and partially sighted people are living in social housing including sheltered housing accommodation, compared to 12% of the overall population (Russell 2013; NIRSA 2013b, May). LTHS are now mandatory for all new social housing in NI. Although LTHS enable people to remain independent in their homes for longer (Hanson 2001), previous research suggests that LTHS focus more on the needs of those with physical impairment than the requirements of people with sensory impairment (Holland and Peace 2001; Imrie 2004b; Madigan and Milner 1999; Milner and Madigan 2004). Furthermore, studies indicate that building users should have greater input into creating and improving future design standards (Imrie 2006; Milner and Madigan 2004; Percival and Hanson 2005).

The quality of the home environment influences an occupant’s mental and physical wellbeing, although not always positively (Imrie 2004a; Iwarsson 2005; Jackson and Kochtitzky 2010; Jackson 2003; Werngren-Elgstrom et al. 2009). Home is many things; it is a container of wellbeing, a place of security, a space where social life, leisure and recreation take place (Bachelard 1964; Stretton 1976). It is also part of a self-identity, built up over time around memories using many senses, including sight (De Botton 2006; Pallasmaa 2005). Cultural security, identity, relationships and mental capabilities are formed at home (Lewin 2001). Heidegger (1993) maintains that home is an integral part of human experience because individuals engage with it in everyday life. Therefore, taking the lived experiences of housing occupants into consideration is crucial when designing homes, especially as dissatisfaction can result if human needs or motivations behind home-making are ignored by designers (Zavei and Jusan 2012).

Imrie (2004a) asserts that many disabled people are not experiencing several of the concepts of an ideal home. This occurs as a result of poor floor plans alongside design conceptions that do not consider impairments. Likewise, descriptions of “ideal homes” disregard elements of domestic life such as impairment, illness or disease (Chapman and Hockey 1999). Gilman (2002) believes that a home is a human institution and is therefore open to improvement. Besides, Imrie (2004a) notes that, whilst houses may offer elements of an ideal home at a given time, they are transient and could change when people develop impairments. It is therefore important to design homes that can accommodate the onset of impairment in order to maintain some of the concepts of an ideal home in an occupant’s life. Tinker (1997) suggests that a comprehensive evaluation of homes designed for lifetime living needs to be carried out and this should include the perspectives of their occupants.

Previous research describes the emotional effect of glaucoma in participant’s lives and examines the experiences of older men and women diagnosed with macular degeneration (Moore 2000; Moore and Miller 2003; Wu et al. 2011). In line with this, Moore (2000) finds that many activities of daily living are difficult to accomplish by older woman who have developed sight loss, yet when occurring, they face new challenges with optimism. Moore and Miller (2003) note that older visually impaired men struggled with growing dependence on others and used visual aids to overcome this issue. Despite examining, the experiences of groups of visually impaired users, Moore (2000), Wu et al. (2011) and Moore and Miller (2003), do not focus specifically on participants’ descriptions of lifetime homes.

The Chartered Institute of Housing in Northern Ireland (2002) affirms that the adoption of LTHS for social housing is a positive step and that LTHS are of benefit to all users. Nonetheless, it is 15 years since the last report on lifetime homes in NI (Chartered Institute of Housing in Northern Ireland 2002). To date, research on guidelines for visually impaired people and housing in UK were primarily based on studies carried out in England and Wales (Bright et al. 2003, March; Lewis and Torrington 2013; Percival and Hanson 2005, 2007; Rees and Lewis 2003, 2004). For instance, Bright et al. (2003, March) examine lighting levels in the private homes of visually impaired people. Lewis and Torrington (2013) assess the quality of lighting in English extra-care housing schemes for visually impaired people. They then suggest that that although many extra-care schemes provide adequate lighting levels, illuminance could improve in lounges and bedrooms. Percival and Hanson (2007) interview visually impaired residents in London. Their research suggests that tenancy documents often have inaccessible formats for visually impaired people and that there is a need to improve housing allocation procedures. Hence, it is necessary to examine lifetime homes of visually impaired people in NI to begin to determine their effectiveness.

The aim of this research is to therefore examine the experiences of visually impaired people living in lifetime homes in Northern Ireland. More specifically, the main objective of this study is to investigate the suitability of LTHS for visually impaired people.

## Methodology

This paper examines the experiences of visually impaired people living in lifetime homes through interviews;

- to investigate existing lifetime homes,
- to identify whether LTHS met their needs, and
- to better understand their experiences.

### Procedure

A phenomenological approach was undertaken to obtain an understanding of first hand experiences of building users with visual impairments. Interviews followed a semi-structured schedule in keeping with guidelines by Kvale and Brinkmann (2009). This allowed for flexibility and ensured that questions were as neutral as possible. Questions were open-ended and included topics of home, housing selection, accessibility and circumstances of participants’ visual impairment. The schedule was tested and trialled using pilot interviews allowing conditions such as timing to be examined (Arksey and Knight 1999; Turner 2010). Interviews were conducted in participants’ own homes to ensure that they felt confident and at ease. Interview schedule questions included the following:

- Can you tell me about your Visual Impairment?
- Could you describe your current home?
- Can you describe how important or unimportant your home is to you?
- Can you describe how you chose your current home?
- Can you describe how your home makes you feel?
- In your experience, what part of the home is most important to you?
- Can you think of any advice for house designers in the future?

### Participants

A homogenous sample of participants was used for this study as is common with IPA research (Smith and Osborn 2008). This also facilitated the evaluation of the transcripts of multiple interviewees in a similar context. Access to home user samples was gained through gatekeeper Housing Associations (HA) administrative databases, through charity organisations and snowball sampling. HA are voluntary organisations that aim to help people access affordable accommodation that meets their requirements and became the main providers of social housing in the UK following the Housing Act in 1988 (Page 1993). Currently, there are 22 HA in NI. The details of HA home occupants are contained in confidential administrative databases or lists that are accessed and managed by individual HAs. As HA administrative databases are confidential, researchers do not have access to the personal details of HA tenants. Therefore, letters to potential participants from the database were sent by HA officials who acted as gatekeepers. McFadyen and Rankin (2016) maintain that gatekeepers have a responsibility to ensure that occupants remain protected and can base selection of participants on assumptions. Therefore, to explain the purpose of the research and to reduce preconceptions, meetings were held with gatekeepers prior to selecting potential participants.

Eligibility criteria were therefore based on whether or not residents were registered as partially sighted or blind and whether homes were built to all of the 16 criteria set out by LTHS. Potential participants were asked to describe their impairment and a checklist with LTHS criteria was used to ensure compliance with standards. Six participants with a mean age of 59 were interviewed as outlined below (Table 2):

**Table 2:** Sample

Participant number	Age	Type of visual impairment	Other medical conditions	Registered level of visual impairment	Years since registration	Living alone or with others	Gender	Marital status	Rural/urban area	Tenure type	Occupation	Building type
L1	82	Glaucoma	Arthritis	Partially sighted	12	Alone	Female	Widow	Urban	Social housing	Retired	Apartment
L2	45	Retinal detachment	Diabetes	Blind	7	Alone	Male	Single	Urban	Social housing	Unemployed but previously worked in construction	Apartment
L3	43	Congenital glaucoma & corneal damage	Diabetes	Blind	43	With others	Female	Single	Urban	Social housing	Unemployed	House
L4	47	Diplopia	Poor hearing, asphyxia as a result of an acoustic neuroma	Partially sighted	10	With others	Male	Single	Rural	Privately owned	Unemployed but previously worked in the public sector	House
L5	83	Age related macular degeneration & one eye removed due to tumour	None	Blind	33	Alone	Female	Widow	Urban	Social housing	Retired	Apartment
L6	51	Blind in left eye and peripheral vision only in right eye	Aphasia	Blind	4.5	With others	Male	Married	Urban	Social housing	Unemployed previously self-employed	House

### Data analysis

Interviews were analysed using interpretative phenomenological analysis (IPA), which is a systematic qualitative approach that examines the individual lived experiences of participants (Osborn and Smith 2006). IPA was deemed a suitable method as it allowed for deep descriptions of how participants thought and felt about challenges that they experienced in the home, whilst acknowledging that individual researchers may bring certain concepts during the analysis process (Reynolds 2003; Smith et al. 2006). It was identified as an ideal technique for in-depth analysis of small sample groups and each interview was analysed through a series of six steps of IPA as devised by Smith et al. (2009). This included initial reading, noting, developing emergent themes, searching for connections, moving to the next case and looking for patterns across cases.

Interviews were recorded and transcribed verbatim with wide margins to facilitate note making during analysis. Non-verbal utterances, for example a pause or laughter, were indicated in brackets (Smith et al. 2009). Transcripts were read multiple times to develop an understanding of what was said. Then exploratory comments about language, description and conceptual notes for each interview were developed. A research diary was kept to record initial thoughts of the researcher for bracketing purposes. Potential themes were identified and grouped according to apparent meanings and relationships to each other (Shearing et al. 2011). Transcripts for each individual interview were re-read to ensure that verbatim data supported themes (Handley and Hutchinson 2013) and finally super-ordinate themes were developed across interviews.

### Quality assurance

Guidelines for enhancing quality of research were implemented by the research team (Elliott et al. 1999; Yardley 2000). Thus, an audit was carried out by the second author to ensure that coherent links existed between interpretations and original transcripts (Handley and Hutchinson 2013). In addition, transcripts and themes were discussed with independent researchers to review data for discrepancies. Transparency of the decision-making process was maintained by using a research diary, and each step of the analysis process was recorded using tables. Credibility was strengthened through the use of direct quotes in the results section of this article.

## Results

**Fig. 1:**
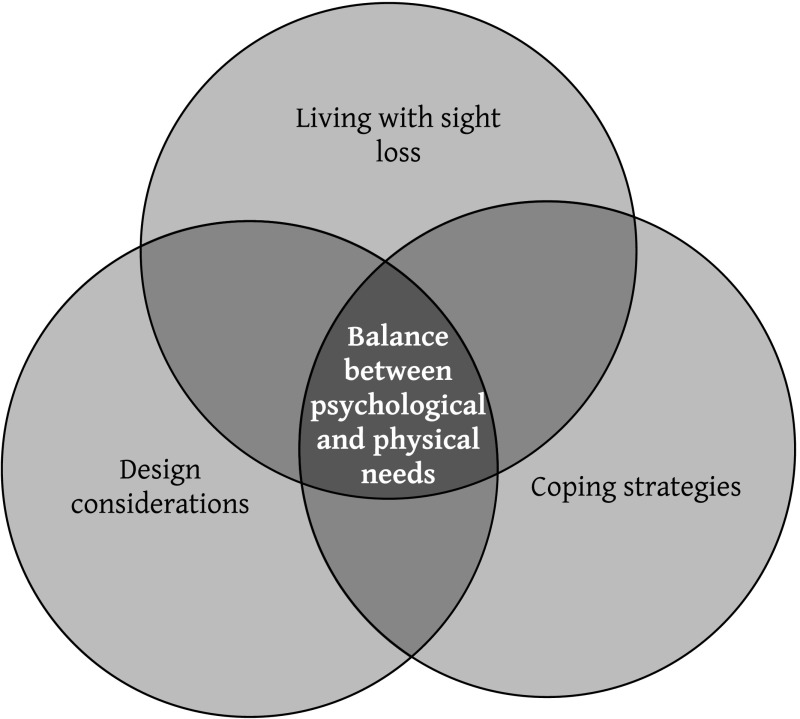
Schematic representation between identified themes

Three super-ordinate themes emerged from analysis: (1) experience of sight loss, (2) design considerations and (3) coping strategies. Super-ordinate themes interconnected forming a core theme of balance between psychological and physical needs (Fig. 1; Table 3).

**Table 3:** Super-ordinate and sub-ordinate themes

Super-ordinate theme	Sub-ordinate themes
3.1 Living with sight loss	Negative consequences of the diagnosis
	Individual needs
	Challenges in the neighbourhood
	Challenges in the home
3.2 Design considerations	Meaning of home
	Implications of positive building design features
	Implications of negative building design features
	Lighting needs
	Improving lifetime homes
3.3 Coping strategies	Positive emotional response to sight loss
	Familiarity
	Physical ways of coping
	Support

### Living with sight loss

Living with sight loss was conceptualised into four sub-ordinate themes: challenges in the home; challenges in the neighbourhood; negative consequences of the diagnosis and individual needs (Table 3).

#### Negative consequences of the diagnosis

All participants associated visual impairment with negative emotions. Participant L1 expressed a sense of helplessness with the loss of her eyesight: “But it’s just…they just closed down on me and that’s it. There is nothing that I can do about it”. She felt she could no longer rely on her vision. Accordingly, she reported that she was anxious and self-conscious outside her home. She coped by shutting her eyes in public and placing her hands over her face and tried to avoid stairs as much as possible:

I don’t be anywhere there are stairs. There’s elevated steps and whatever…You get a wee glimpse you know here and there, keeps you going. It keeps you from walking into things (L1).

Participant L2 too experienced negative emotions: “Well it definitely didn’t make me feel too good…but I just had to get on with it.” The words “had to” suggested that he felt he had no choice. When later going on to describe his sight loss like being in “the black hole of Calcutta,” implies that he felt trapped. His diagnosis also depressed him: “Emotionally I was all… I was just gone”. After his diagnosis and loss of independence, he had to downsize and adapt to new surroundings, compounding his anguish. “Was I hurting for the new house or the fact that I had to leave the old house…it’s a hard one to say really” (L2).

Participant L4 expressed upset following his diagnosis, not just because he needed to be more careful outside his home but also because he could no longer read, something that he previously enjoyed doing. Similarly, when Participant L5 had her right eye removed, she reported feeling numbed by the experience:

So that left me with very little vision at all, because that was my good eye. And that had been removed…..um….it all happened so quickly that I didn’t feel that I had time to feel anything (L5).

This theme therefore highlights some of the participants’ negative experiences in addition to their physical and emotional needs at home. Frustration and a sense of loss of both sight and independence were commonly expressed.

#### Individual needs

It was apparent that no two participants’ needs were ever the same. Whilst some participants lost their sight suddenly, this was more gradual with others. This meant that participants’ needs sometimes changed with time, as evidenced when Participant L1 reported that she required less space when now living alone: “I told them that I didn’t really need two bedrooms now because there was only myself” (L1).

Participants also sometimes presented with more than one disability, adding further complexity to just considering the challenges presented by visual impairment alone. Participant L4, (who commissioned his house be built to LTHS) and who was also challenged by deafness and physical impairment recognised this when stating; “when we came to design the house, the sight and all was in the mix… a part of the mix with me” (L4) before going onto explain that compromises then had to be made stating “If I didn’t have other disabilities you would have been able to concentrate far more on …maybe different floor types” (L4). Hence, there will be times when one design solution will not best address a person’s needs. For instance Participant L6, who had physical impairment and stated that he felt “the loss of sight more than that of his mobility” (L6) might then have favoured more emphasis on that aspect in addition to ensuring the 16 criteria of LTHS. Thus, it is important to ensure that designers do always try to better understand occupants’ physical and psychological needs.

#### Challenges in the neighbourhood

All participants expressed that they were happier in their homes than being outside in their surrounding neighbourhoods and often described difficulties experienced outside their homes:

Well I mean I can only speak for myself but…um…I’m fine in the house but…once I would go outside my own gates…um…if I don’t have the dog with me I’m completely lost (L3).

Participant L1 described a fear of accidents outside her home and therefore ventured out alone, “only when she had to”. Then her eyes would sometimes shut uncontrollably due to stress: “… yesterday I was up the road I had to go up to Tesco and I was coming back down again. I just, just…couldn’t see” (L1).

Repetition of the word “just” in the above extract suggests her frustrations and fears of being alone or lost outside her home. She, like Participant L6, preferred to leave home with the help of family members. Participant L3 even described how her dog had to walk onto the road due to parked cars on the footpath adding to her sense of unease.

These shared challenges, both physical and psychological, highlight the comfort and value of the participants’ own homes as places of safety, refuge and rest separate from the world outside their front doors.

#### Challenges in the home

Negative building features sometimes had major implications on the quality of life of participants. This could involve the very basic action of access in and around the home. For instance, participant L3 disliked her home’s hilltop location in winter, as she was then socially isolated and confined indoors due to snowfalls: “In the winter time it’s not an easy place to get in and out of” (L3). Moving to inside the home, some participants voiced concerns with regard to the demands of extra wide doorways required to ensure wheelchair access in the LTHS. Participant L4 expressed that power-assisted doors were too expensive to install, stating that: “I didn’t go for the high tech solution for a few reasons. First of all they’re expensive and I really do think that they are just a big label; their manufacturers tend to put the hand in and make them expensive” (L4). Instead, manual doors were installed. This fact was, however, problematic for Participant L5 who expressed her difficulty with the larger manual doors stating: “But as you get older obviously…the doors are very heavy…so if you hold onto the door, if you leave it to close by itself, it would nearly wreck the whole apartment” (L5). She was aware that leaving the door open with an object could be dangerous in a fire or be a trip hazard and so instead often had to ask others to open doors for her. This fact suggests that the conflict between fire safety and internal doorway size should be addressed for the elderly population.

Problems with access also extended to getting to and then reading heating controls in the home. Participant L3 expressed this stating: “…well in here…um…they have…like…a digital display on the oil heating. Well I can’t see it to read it” (L3). Participant L5 was also unable to see the code for her electric or gas meters and was unhappy with the positioning of meters in her home explaining, “They are all down in corners. Awkward…” (L5).

This affected their independence as both participants reacted by asking sighted family members to input information into meters for them. It also afforded less choice to Participant L5 as she eventually decided to pay for her electricity through direct debit to alleviate the need to check her meter on a regular basis, but found this strategy unfortunately more difficult to budget for.

### Design considerations

#### Meaning of home

All participants had an emotional attachment to their homes and there was a common consensus that home was for each, a secure place. Participant L1 stated that home was “everything” to her and she felt most confident there, as it was familiar to her. She was proud of her home and was determined to keep it clean despite the associated challenges in doing so. It was for her, a base and a place of refuge and for her:

…Very important, because you need shelter for a start…somewhere to be based and somewhere to concentrate on with the disability that you’re so surrounded with blindness and really that’s…just…you need like a foundation and especially that is what my home is now - a foundation (L1).

The location of the house and knowledge of the home’s surroundings was also considered significant. Participant L5 moved back to one of her previous residential locations for this reason: “everybody kind of knows me, even now like with living away” (L5). Likewise Participant L2 wished to return to a familiar environment as he disliked living away from his family; “Well I would probably love to live, you know, in a wee bungalow…where I was from” (L2).

Participant L5 was considerate of her family’s needs when choosing her home, wanting to live near them and to have a view of the mountain, even though she could no longer fully appreciate it, suggesting the importance of both people and place when considering the idea of home.

All participants described their homes as places of comfort. Participant L2 believed it should provide security and shelter without feeling institutional as he lived with people that had no disability. Equally, Participant L6 stressed the importance of companionship stating that he disliked it when people left the room that he was in. The importance of the home was a continuous theme throughout analysis as participants were long-term residents in their homes and considered their homes as safe havens, also as a place where they felt most comfortable and confident. Though that was sometimes not without worry. For instance, Participant L3 reported worrying about safety at home, having the responsibility of two young children: “…well safety for me would be a big issue…with me not having any sight” (L3). Whilst for security, Participant L4’s home was on a flat site eliminating the need for a ramp, thereby purposely disguising the fact that it belonged to somebody with a disability. So a sense of vulnerability could still be evident for residents, despite the implementation of the LTHS.

#### Implications of positive building design features

Participants reported many benefits to living in their LTHS accommodation. Ease of access within the homes was a recurrent theme. Specifically Participant L1 moved to a LTHS apartment from an apartment in an isolated area for that reason. Participant L2 was satisfied with a simple design excluding steps in his home: “I’m very happy with that because it is straightforward” (L2). Participants L4 and L6 chose a single storey house whereas, Participant L5 was satisfied with a first floor apartment that was built on one level.

Participant L6 also stated that he appreciated the benefit of wider doorways for his visual and physical impairments. That additional space, provided with wheelchair access in mind by the LTHS, was also valued by participants. Participant L2 moved to a lifetime home for the extra space needed to house his belongings, for example, his quadrant, computer, Braille machine and guide dogs. Participant L6 also appreciated that the extra shower room that accommodated his wheelchair could also be used by other able-bodied members of his family. Participant L5 also reported enjoying shower accessibility in her home, whilst Participant L3 was happy to use her downstairs shower room to store her children’s bicycles.

Some participants also appreciated the LTHS stipulated socket and switch heights. This was voiced as being beneficial by Participant L3 “You know the switches, the light switches and everything else…It’s good because you are…kind of…not feeling around the walls to find out where they are or anything like that” (L3).

**Fig. 2:**
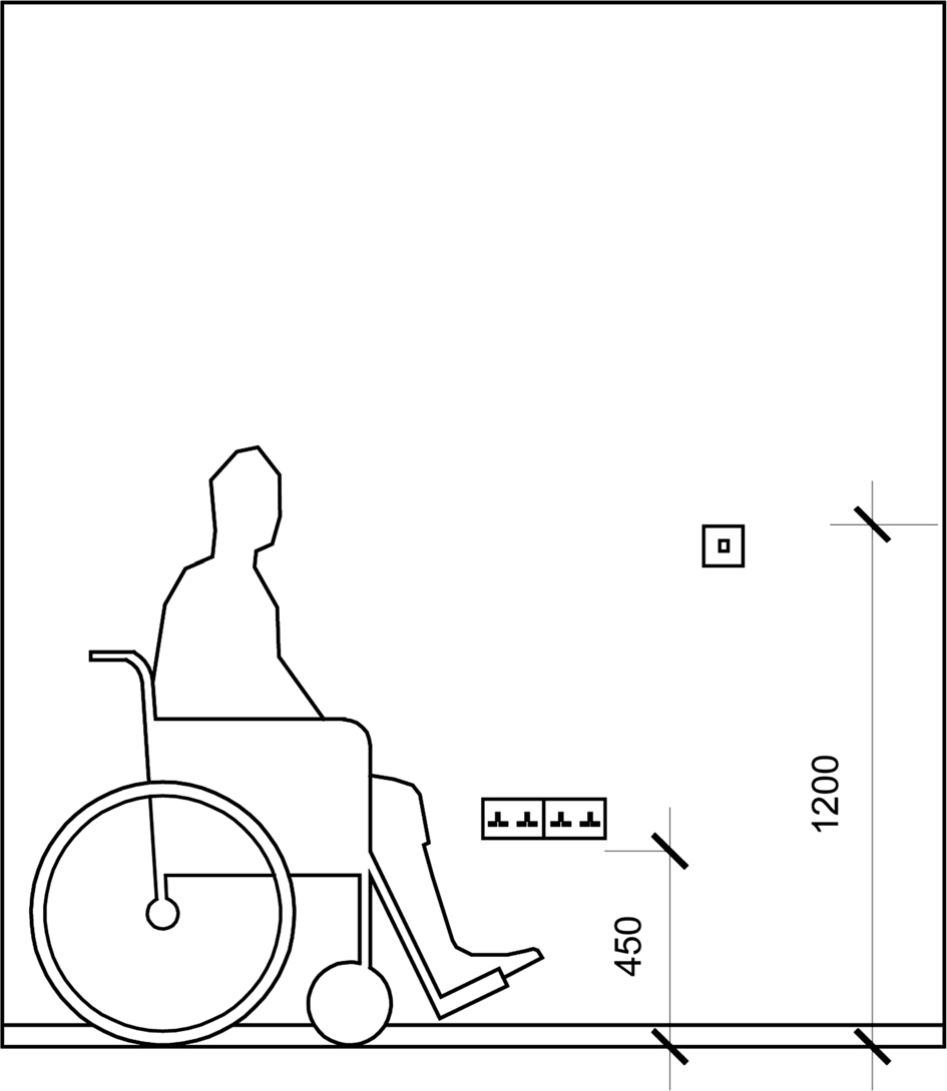
LTHS guidance for location of switches and sockets

This demonstrates that an LTHS guideline outlining correct switch height, originally designed for those with physical impairments, can be of benefit to others (Fig. 2). Similarly, Participant L4 praised his switch and socket heights before praising the provision of a car port as this kept him dry: “the carport has been a very good thing for disabled people; ‘cos…you haven’t to rush to get into your car if it is raining or anything like that” (L4).

Thus many LTHS features added positively to participants’ lives. Wide spaces were important for both visually and physically impaired people with participants also appreciating when their homes had no steps and the extra space that their homes afforded. They were also able and willing to find alternative uses for some of the LTHS features used also by other able-bodied residents indicating once again the challenge in providing one set of design criteria for all residents.

#### Implications of negative building design features

Despite the well-intentioned nature of the LTHS, participants still reported negative aspects of their homes. These were in relation to not having direct access to the rear garden from their house, room layout and dissatisfaction with internal fixtures and fittings.

**Fig. 3:**
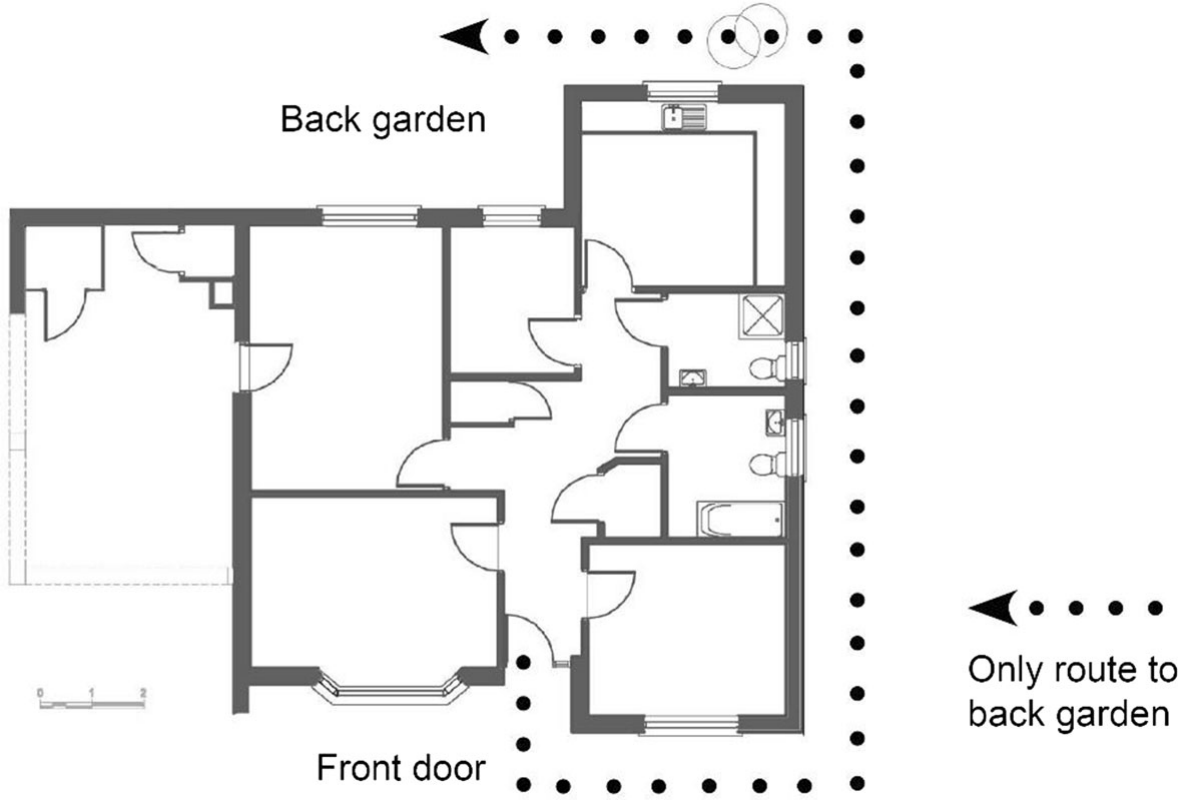
Diagram depicting poor access to L3 back garden

Participant L3, who had young children, and whose house did not have back door access, had to access her garden via the side of her house (Fig. 3).

Accordingly for safety, Participant L3 kept her guide dogs in the front carport to deter intruders as she worried about leaving her children alone in the house or garden stating that “you have more control over your back garden and what’s about and where you are putting your feet than…..walking out on the street…if the youngsters had been playing at the side of your house you don’t know what you are walking into” (L3).

Participant L4, who did have a back door, was unfortunately unable to open it fully due to an access ramp that was built to minimum access standards.

Whilst many participants were satisfied with the amount of space that their homes provided, Participant L2 felt that there was not enough space and suggested an extra bedroom for visiting family members: “Even though I might be single…I would like to have two rooms… having family to come round and see me, if you were ill for whatever reason…instead of having to go to sleep on your settee or your living room”(L2).

Participant L6 felt that he would benefit from an en-suite bathroom because his main bedroom was a large distance away from his shower room and the shower was too small to accommodate his carers when he needed aid: “The way it was laid out it…um…was making it difficult for somebody to be in there…you know because they would get soaked” Compounding the situation, the absence of windows in the shower room, (as was also the case for Participant L3), led to internal condensation despite the use of an extractor fan.

Internally, a number of the participants felt that the universal nature of the LTHS was not individual enough for them and their own specific situation. For instance, although kitchen worktops were adjustable to cater for different physical needs in her home, Participant L3 did not use that facility and she was dissatisfied with her kitchen windows:

The window in the kitchen as well…I can see their point coming from probably somebody in a wheelchair’s perspective…the big long bar but, if you want to put say…a louvre blind or something like that up on the windows, you can’t do it (L3).

Practical design and functionality were important for a variety of impairments. Participant L4 did not want his kitchen designed to focus solely on disability, as the other occupants of his home were able-bodied. He went on to state that he believed the needs of individuals should still be considered within universal design:

I think there should be universal design but, then you need to come down to a specific person, a specific client. I am actually quite tall….so I find the standard rails that they have, are terribly low (L4).

Further emphasising the individual likes and dislikes of residents, some discord was voiced over the number and position of electrical switches and sockets. For instance, Participant L4 wanted more power points in his home and felt that the switch for his bathroom fan was too high. Also, his home lacked a pull down system to retrieve dead batteries from his smoke alarm. Participant L5 was unimpressed by set height switches and wider doors in her home: “Um… I think they are just…they are fine, they are adequate” (L5). Using the words “fine” and “adequate” suggested that the standards were acceptable but not to her liking.

#### Lighting needs

Whilst lighting had no impact on Participants L2 and L3, as they had no sight, the other participants frequently mentioned the quality of lighting throughout their narratives indicating how important it was to them. Notably, lighting was used to create a comfortable atmosphere in the home irrespective of visual impairment. Again, however, lighting needs varied between individuals suggesting that there needs to be more consideration given to satisfying the individual needs of different people as regards design in the home.

One lady disliked light and used blinds to limit light in her home: “I don’t like the light. I don’t put that light on” (L1). Participant L6 used blinds to prevent glare. Participant L4 felt that lighting levels needed to be increased and was dissatisfied with the quality of light in his bathroom: “Well, I made a few mistakes with the house I think. Not to bring in enough natural light really, well it’s free for one thing. I like the sense of light in the hall” (L4). He rarely used dimmer switches and automatic lights in his hallway would have benefited him “I’d love to have something…like…that comes on as you move through the area” (L4). His energy efficient light bulbs, whilst efficient produced a poor quality of light and were described as being dull: “It’s such a huge, huge problem. I worry about it every day” (L4). Equally, Participant L5 felt that her light bulbs were not bright enough for her needs and wanted florescent lighting in her kitchen. So whilst energy efficient light bulbs have benefits, the priority for those with visual impairment appears to be the quality and strength of the light in addition to choice.

#### Improving lifetime homes

**Fig. 4:**
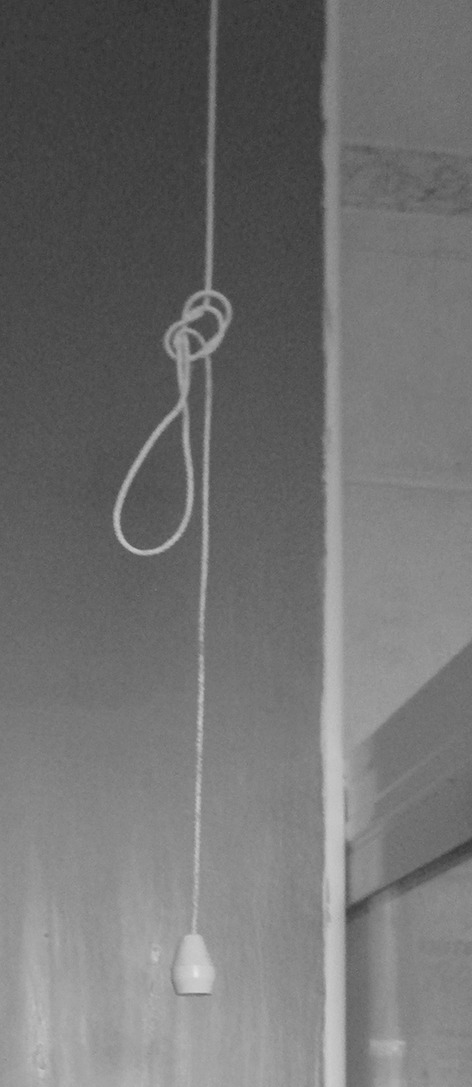
Shortened pull cord

For participants, tidiness and organisation helped to prevent against falls. To aid in that, provision of adequate storage is beneficial. All participants liked company at home preventing feelings of isolation and most had pull cord care-line systems installed at home to alert help in emergencies (L1, L2, L3 and L6). However, Participant L6 shortened the cords to keep them out of his grandchildren’s’ reach (Fig. 4):

Participant L4 felt that it was important to consult with designers when planning their homes:

Definitely you know, you do need the architect who knows to put the things in…you definitely do. But… the architect he doesn’t know our lives really….so it takes a combination of the two… (L4).

He favoured under floor heating recounting the fact that radiators acted as obstacles in his previous home. This once again illustrates the importance of designer-awareness of the considerations for visually impaired people and the importance of user involvement at an early design stage.

### Coping strategies

Strategies employed to cope with visual impairment in the home were a recurrent theme in transcripts and again the balance between psychological and physical needs was embedded within this theme (Fig. 1).

#### Positive emotional response to sight loss

Occupants of Lifetime homes used acceptance to cope with challenges: “So, I just have to get on with it. It’s like everything else you get used to it” (L1). Using the words ‘have to’ indicated an absence of choice. Nonetheless, participants had a positive outlook and were determined to continue with their lives: “…aw, I don’t groan and moan over it…its good days and bad days, same as everybody else” (L1). Participant 1 also compared herself to others in more difficulty: “there are people worse off than I am” (L1). Participant L3 focused on positive thoughts when separated from family after moving house: “but at least they were always on the other end of a phone” and coped practically by asking others to turn on any complicated heating controls: “I would get them to work it for me. It was the same in my last house as well” (L3).

#### Familiarity

Following diagnosis, participants had to adjust by re-assessing their homes:

I don’t touch it, I just feel it. And I know that is the voice monitor it would be on my right hand side, and sometimes…you know…if I touch it when I go to my left hand side, that’s the door in, and then again I just walk across until I feel another corner of the wall and that’s, basically when I sit down (L2).

This process of familiarisation requires good memory to recall where guides are positioned and may be referred to as mind mapping. This process continued for a number of months once participants moved into their homes: “how I coped with the new house I lived in was…I basically just practiced and practiced and practiced” (L2). This involved learning where obstacles were located and sometimes even could result in minor injuries for participants: “I kept walking into that wall” (L1). Knowledge of a home’s layout prior to diagnosis made it easier for participants to produce a mind map of their home with gradual vision loss. This familiarity gave participants a sense of comfort and confidence that they did not experience in unfamiliar spaces. Although this theme did not relate directly to the design of lifetime homes, the importance of the home as a place of familiarity and comfort should be noted.

#### Physical ways of coping

Participants coped by adding or changing physical elements in the home, for instance one man used existing products in a different way:

You might have noticed this; the windows are actually upside down. And why I have done this, is so that the handles are on the bottom. So I can actually open and close the windows. If they were at the top I couldn’t reach them at all. So its wee small things like that (L4).

He also had a physical impairment that influenced his decisions on adapting his home and used fire doors as these were the only wide-swing doors available. Participants L3 and L4 felt colour contrast around steps and light switches were important for partially sighted people and they altered their homes using tactile stickers: “Um…well, on my oven I would use…wee markers, wee bump-ons” (L3). To tell the time, one participant’s clock chimed every fifteen minutes and one lady used bells on her guide dogs to detect them in the home. Tenants needed support to make changes and improve elements of their homes. However, requested changes were sometimes opposed by HAs: “Um…I have spoken to them a few times, about that. I have got letters sent in from social workers and support workers and stuff and just, nothing ever seems to materialise from it” (L3).

#### Support

Whilst participants wanted to remain independent, they were assertive enough to seek help when required with support from family and HAs being important to them. One participant’s daughter took her shopping as the journey was challenging when alone: “I mean, I go for messages; my daughter comes in a car and would take me” (L1). She also received additional support from the HA. Another participant felt that visually impaired people should have confidence in seeking help: “If you have a disability it is going to be harder for the disabled person, but, they need to find help” (L2). Participant L3 relied on her children to control the heating in her home, another depended on her daughter for support: “My daughter, she comes up at lunch time and…um…makes me some lunch…and maybe organises a dinner” (L5).

It was clear that participants used coping strategies relating to physical and psychological needs and examining their coping strategies contributed to understanding experiences of being visually impaired.

## Discussion of findings

The housing experiences of visually impaired people living in Lifetime Homes were identified. As with previous research showing that agency^2^ is limited by unexpected movement or clutter at home, trip hazards were present in lifetime homes but were mainly due to human activity (Allen 2004). Currently criterion 16 of LTHS contains recommendations for switch or socket height and tonal contrast on controls. These, whilst not mandatory, are good practice recommendations (Lifetime Homes 2010). Positive aspects of Participant’s Lifetime homes include simple design, level access and no steps, which correlates with Hanson (2006) who recommended simple layouts in the homes of visually impaired people.

This study also shows that visually impaired people place an emotional value on their homes, reaffirming Heidegger’s theory that a home is not just an object to admire, but it influences human experience (Heidegger 1993; Sharr 2007). In line with the work of Bachelard (1964), De Botton (2006) and Pallasmaa (1994), visually impaired people identify the home as a place of security and comfort. Housing satisfaction is also dependent on a home’s location as it strongly influences its occupant’s approval (García-Mira et al. 2005). Furthermore, location is fundamental to wellbeing in supporting independence and preventing isolation (Goodman 2008; Hanson 2006).

This study’s findings concur with previous research and show that some occupants feel isolated due to their homes’ locations. This may be explained by the fact that they have a level of control at home and are more likely to encounter unexpected hazards outside (Allen et al. 2002).

De Leo et al. (1999) state that there are four reactions to a sight loss diagnosis: acceptance, denial, depression and anxiety. Our findings corroborated those from previous qualitative studies, highlighting that negative consequences of diagnosis include lack of control, feeling depressed, anxious or self-conscious (Nyman et al. 2012; Wu et al. 2011). Riazi et al. (2012) agree that individual needs and capabilities should be considered, however, for occupants with multiple impairments, it is difficult to differentiate between visual and physical challenges.

This research study determines that, as an individual’s needs change, they experience a new need for support and some expressed that moving home is stressful. Evidence shows that moving home is a stressful event requiring support (Percival et al. 2006). When choosing a home, occupants take their family’s needs into consideration and wish to live in familiar areas. Previous studies also demonstrate that support cannot only help someone adapt to a new environment, but can ease negative consequences of diagnosis (Duckett and Pratt 2001; Girdler et al. 2008; Moore 2000; Moore and Miller 2003). Preceding work also suggests that sometimes visually impaired people can experience isolation and are at risk of social exclusion therefore support is an important aspect of their lives (Barnes et al. 2012; O’Neill 2003; Percival and Hanson 2005).

Allen et al. (2002) state that barrier-free housing is often built to poor space standards with no allowance for the equipment or technology used by some visually impaired occupants. However, participants who live in lifetime home in the current study relocated to attain extra space. This finding is also in contrast to research that explores space standards in the UK for the general population and states that home occupants were dissatisfied with storage space (Drury 2009; Finlay et al. 2012; Roberts-Hughes 2011). Benefits of LTHS for occupants are sliding doors, carports and higher switches. In addition, views from residences to connect with the outside world are important for partially sighted people with less mobility. Research on guidance also discusses the need for provision of views of natural features, dynamic urban scenes, and reasonable views to prevent isolation (British Standards Institution 2008; CIBSE 1997; Lifetime Homes 2010; The National Affordable Homes Agency 2008). A paucity of windows is oppressive for occupants (Hanson et al. 2002).

Negative aspects of lifetime homes include poor access outside the home with ramps that can be built to minimum standards that are inadequate for individual participants’ needs. Some find that kitchen design and lower windows are not useful for someone with visual impairment. However, participants viewed lower level smoke alarms as beneficial, indicating again the benefits of designing to meet individual needs. Criterion 6 of LTHS requires wider doorways; however, wide internal fire doors are sometimes too heavy for occupants to open. Goodman (2008) suggests that internal doors should be of a sliding nature or opening into the room with the leading edge against an adjacent wall to prevent accidents. Although Criterion 14 of LTHS discusses bathroom design, it does not include ventilation which in this study was sometimes found to be inadequate. This may be because lifetime homes are often designed to allow homes to reach liveable standards in order to be fully universal, by primarily focusing on meeting the needs of wheelchair users (Milner and Madigan 2001). However, despite this, some shower room spaces were described as too small to facilitate carers, indicating that minimum standards are still not good enough.

Good quality lighting is particularly important for visually impaired people (Bright et al. 2003; Brunnstrom et al. 2004). Also, partially sighted occupants are unhappy with energy efficient and florescent lighting in their homes. Under EU regulations, governments are phasing out inefficient lighting (The Commission of the European Communities 2009) and whilst sustainability is important, it is necessary to ensure that replacement lighting is sufficiently bright. There is scope to carry out future research to determine a suitable yet environmentally friendly alternative. An environment can be perceived as positive by one individual yet considered to be negative by another (Harrison and Tweed 2006, November). Thus, occupants’ lighting needs can vary. Fisk and Raynham (2014) maintain that adjustable lights can be introduced to match individuals’ preferences therefore adjustable lighting needs to be considered in current guidelines.

Occupants suggest improvements to LTHS such as greater consultation between users and designers, use of colour and the implementation of under floor heating to eliminate the need for obstructive heaters. Stone (2008) states that older people particularly appreciate space to accommodate guests and this study supports this finding whereby, in the case of a single blind occupant, an extra bedroom was needed to host visitors.

Coping strategies were discussed to further understand experiences of visual impairment. Gibson describes affordance as that which an environment offers a person for good or for ill (Gibson 1978). Participants respond to negative affordances such as clutter by adapting their homes to minimise negative effects. They also use sound and tactile stickers to adapt homes. In keeping with previous research, occupants focus on positive thoughts and receive support from friends (Moore and Miller 2003). Occupants also study new and unfamiliar environments by using touch, developing mind maps and memorising where obstacles are located. See Table 4 where implications and findings of this study are summarised:

**Table 4:** Implications and findings

*What is already known about this topic*
Visual impairment is increasing
Lifetime homes aim to make homes more accessible
Previous research has not focused on the lived experience of visually impaired occupants of lifetime homes
*What this paper adds*
An insight into the experiences of visually impaired people living in lifetime homes
Criteria 14 and 16 make need to be revised to take digital controls alongside ventilation into consideration
Occupants appreciate the extra space afforded by lifetime homes
Residents regard their home as a place of security and comfort
Negative design features limited occupants’ capacities to maintain independence.
*Implications for practice*
Consideration should be taken into the location of lifetime homes at design stage
Future architectural research would benefit by using IPA
Further research is needed to confirm changes to criteria 14 and 16
Future research should consider the use of post-occupancy evaluations to compliment qualitative research

## Limitations

Many HAs did not have detailed records pertaining to occupants’ impairments, thus limiting access to LTHS occupants and contributing to the small sample group. Although a small sample size limits generalisation of the findings, interviews provided a rich and in-depth account of participants’ experiences. IPA was used to analyse data, as it is suitable for in-depth analysis of small sample groups. Polit and Hungler (1995) maintain that qualitative analysis procedures rely on subjective judgements that may limit generalisability; therefore, quality checks were implemented to reduce subjectivity as described in Sect. 2.4. Participants sourced through charity groups for blind people may have better access to housing aids.

## Conclusion

This study has provided a useful understanding of living in lifetime homes. However, previous studies demonstrated that people suffer from a lack of fluency when discussing space as a topic (Franck 2009). Furthermore, there may be discrepancies between what visually impaired people say about their experiences and how they actually experience it (Herssens and Heylighen 2010). Thus, post-occupancy evaluations^3^ may be beneficial in overcoming these limitations. Nonetheless, this study contributes to scientific literature by offering a better understanding of living in lifetime homes with sight loss, with findings supporting earlier studies that suggest the LTHS need to do more for people with sensory impairment and that there is a need and a desire for individual building users to have more input into the design of their own homes. Finally, as architecture often focuses solely on aesthetics, future research would benefit by using IPA to help provide a deeper understanding of residents’ needs for designers.

## Footnotes

1 Housing provided by government and non-profit groups to people on low incomes. There are over 44,000 social housing units in Northern Ireland (Altair 2014).

2 Action or intervention producing a particular effect.

3 A post-occupancy evaluation is a systematic way to evaluate critical aspects of a building’s performance (Hadjri and Crozier 2009).
